# The Surface-Exposed PA^51-72^-Loop of the Influenza A Virus Polymerase Is Required for Viral Genome Replication

**DOI:** 10.1128/JVI.00687-18

**Published:** 2018-07-31

**Authors:** Benjamin E. Nilsson-Payant, Jane Sharps, Narin Hengrung, Ervin Fodor

**Affiliations:** a Sir William Dunn School of Pathology, University of Oxford, Oxford, United Kingdom; St. Jude Children's Research Hospital

**Keywords:** influenza virus, replication, transcription, RNA polymerase, PA subunit, PA^51-72^-loop

## Abstract

The heterotrimeric influenza A virus RNA-dependent RNA polymerase complex, composed of PB1, PB2, and PA subunits, is responsible for transcribing and replicating the viral RNA genome. The N-terminal endonuclease domain of the PA subunit performs endonucleolytic cleavage of capped host RNAs to generate capped RNA primers for viral transcription. A surface-exposed flexible loop (PA^51-72^-loop) in the PA endonuclease domain has been shown to be dispensable for endonuclease activity. Interestingly, the PA^51-72^-loop was found to form different intramolecular interactions depending on the conformational arrangement of the polymerase. In this study, we show that a PA subunit lacking the PA^51-72^-loop assembles into a heterotrimeric polymerase with PB1 and PB2. We demonstrate that in a cellular context, the PA^51-72^-loop is required for RNA replication but not transcription by the viral polymerase. In agreement, recombinant viral polymerase lacking the PA^51-72^-loop is able to carry out cap-dependent transcription but is inhibited in *de novo* replication initiation *in vitro*. Furthermore, viral RNA (vRNA) synthesis is also restricted during ApG-primed extension, indicating that the PA^51-72^-loop is required not only for replication initiation but also for elongation on a cRNA template. We propose that the PA^51-72^-loop plays a role in the stabilization of the replicase conformation of the polymerase. Together, these results further our understanding of influenza virus RNA genome replication in general and highlight a role of the PA endonuclease domain in polymerase function in particular.

**IMPORTANCE** Influenza A viruses are a major global health threat, not only causing significant morbidity and mortality every year but also having the potential to cause severe pandemic outbreaks like the 1918 influenza pandemic. The viral polymerase is a protein complex which is responsible for transcription and replication of the viral genome and therefore is an attractive target for antiviral drug development. For that purpose it is important to understand the mechanisms of how the virus replicates its genome and how the viral polymerase works on a molecular level. In this report, we characterize the role of the flexible surface-exposed PA^51-72^-loop in polymerase function and offer new insights into the replication mechanism of influenza A viruses.

## Abstract (precis)

Influenza A viruses are a major global health threat, not only causing significant morbidity and mortality every year but also having the potential to cause severe pandemic outbreaks like the 1918 influenza pandemic. The viral polymerase is a protein complex which is responsible for transcription and replication of the viral genome and therefore is an attractive target for antiviral drug development. For that purpose it is important to understand the mechanisms of how the virus replicates its genome and how the viral polymerase works on a molecular level. In this report, we characterize the role of the flexible surface-exposed PA^51-72^-loop in polymerase function and offer new insights into the replication mechanism of influenza A viruses.

## INTRODUCTION

Influenza A viruses are a major global health threat that can cause seasonal epidemics as well as occasional global pandemics (1). The genome of influenza A viruses consists of eight single-stranded negative-sense viral RNA segments which together with the viral polymerase and nucleoprotein (NP) form viral ribonucleoprotein (vRNP) complexes (2). The viral polymerase consists of three subunits, polymerase basic 1 (PB1), polymerase basic 2 (PB2), and polymerase acidic (PA) proteins, and carries out transcription and replication of the viral RNA genome (3, 4). First, viral RNA (vRNA) is transcribed into mRNA by the resident *cis*-acting polymerase of each vRNP, utilizing a cap-snatching mechanism. In brief, the PB2 cap-binding domain binds to the 5′ m^7^G cap of host RNA, which is subsequently cleaved 8 to 14 nucleotides (nt) downstream of the cap by the PA endonuclease domain (5–7). The resulting short capped RNA fragments are subsequently used as primers for viral mRNA synthesis by the viral polymerase. Replication of the negative-sense vRNA is a two-step process. First, an intermediate positive-sense cRNA is synthesized and assembled into vRNP-like complementary ribonucleoprotein (cRNP) structures with newly synthesized polymerase and NP (8). On a vRNA template, replication is initiated *de novo* at residues 1 and 2 of the 3′ end (9) and can be initiated by the resident polymerase of vRNPs *in vitro* (8, 10). Then, cRNA is used as a template for vRNA synthesis and assembly of progeny vRNPs. In contrast to replication initiation on a vRNA template, replication is initiated internally at residues 4 and 5 of the 3′ end of the cRNA template (9). The resulting pppApG dinucleotide is subsequently translocated to residues 1 and 2, where it primes vRNA synthesis (9, 11). *In vitro*-purified cRNPs require *trans*activation by an RNA-free polymerase which does not have to be catalytically active (8). However, the need for a *trans*-acting polymerase in addition to the resident polymerase in replication has also been proposed (12).

Recently, several high-resolution structures of influenza virus polymerases have been obtained by X-ray crystallography and cryo-electron microscopy (cryo-EM) (3, 4). These structures show a polymerase core consisting of PB1, the C-terminal domain of PA, and the N-terminal third of PB2 (13–15). The polymerase core houses the polymerase active site. Protruding from the central core are several flexible domains, including the N-terminal PA endonuclease and the PB2 cap-binding, mid-link, 627, and nuclear localization signal (NLS) domains. These domains have been shown to significantly change their positions relative to each other, depending on the nature of the bound RNA template (13–16). The vRNA promoter-bound polymerase is found in a transcription-ready conformation, which is competent in cap snatching and transcription initiation (14, 15). However, in a cRNA promoter-bound or an RNA-free (apo) state, the polymerase takes up a conformation where the flexible N-terminal domain of PA and the C-terminal domains a of PB2 are significantly rearranged, resulting in a conformation that is not compatible with cap snatching (13, 16).

**FIG 1:**
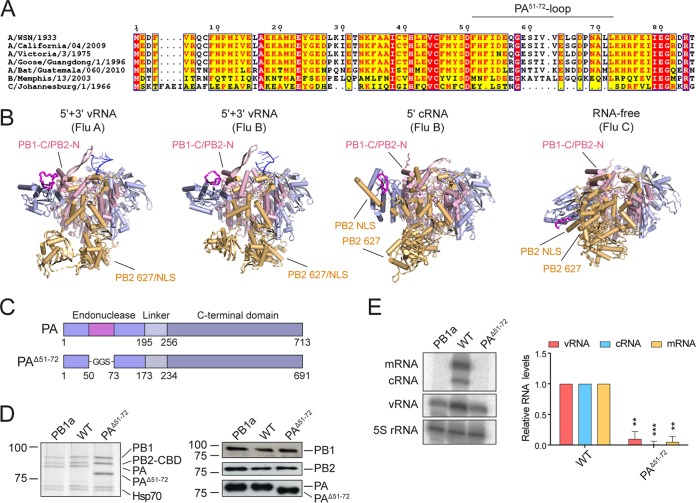
The PA^51-72^-loop is essential for viral RNP activity. (A) Sequence alignment of the PA/P3 N termini of influenza A/WSN/1933 (H1N1), influenza A/California/04/2009 (pH1N1), influenza A/Victoria/3/1975 (H3N2), influenza A/goose/Guangdong/1/1996 (H5N1), influenza A/little yellow-shouldered bat/Guatemala/060/2010 (H17N10), influenza B/Memphis/13/03, and influenza C/Johannesburg/1/66 viruses. Sequences were aligned using T-Coffee (https://www.ebi.ac.uk/Tools/msa/tcoffee/) and visualized using ESPript 3.0 (http://espript.ibcp.fr/). (B) Crystal structures of the vRNA promoter-bound influenza A/little yellow-shouldered bat/Guatemala/060/2010 (H17N10) virus (Flu A) polymerase (PDB accession number 4WSB), the vRNA (PDB 4WSA) and cRNA (PDB 5EPI) promoter-bound influenza B/Memphis/13/03 virus (Flu B) polymerases, and the RNA-free influenza C/Johannesburg/1/66 virus (Flu C) polymerase (PDB 5D98). The PB1 (pink), PB2 (orange), and PA (blue) subunits and the PA^51-72^-loop (magenta) are highlighted as indicated. (C) Schematic of the full-length PA subunit and a PA subunit where the PA^51-72^-loop has been replaced with a GGS linker. (D) HEK-293T cells were cotransfected with plasmids expressing PB1, PB2-TAP, and PA as indicated. Heterotrimeric polymerase complexes were purified by IgG Sepharose chromatography. Purified protein complexes were analyzed by SDS-PAGE and silver staining and Western blotting with antibodies targeting the individual polymerase subunits. Molecular weight markers are indicated in kilodaltons. (E) HEK-239T cells were cotransfected with plasmids expressing the viral polymerase, NP, and segment 6 vRNA. A polymerase containing an active-site mutation (PB1a; D445A/446A) was used as a negative control. Accumulation of viral RNA at 24 h posttransfection was analyzed by primer extension and 6% PAGE. The graph shows the mean intensity signal relative to that of wild-type polymerase from three independent biological replicates (*n* = 3) and corrected for background intensities observed in the negative control (PB1a). Error bars represent standard deviations, and asterisks represent a significant difference in activity from that of the wild-type polymerase (two-tailed one-sample *t* test) as follows: **, *P* < 0.01; ***, *P* < 0.001.

In a previous study, residues 51 to 72 of the PA endonuclease domain were found to be dispensable for endonuclease activity (17). This region forms a surface-exposed flexible loop (PA^51-72^-loop), which has been observed in slightly different arrangements in the structures of the isolated PA endonuclease domains of different influenza A virus strains (5, 18). In other studies, its structure could not be solved due to its unstructured nature (7, 17). Interestingly, in influenza C viruses, this loop is present in a truncated form (Fig. 1A). It has been suggested that the PA^51-72^-loop fulfills a structural rather than a catalytic role in polymerase function, e.g., through mediating interactions with other subunits of the viral polymerase or with a host factor (17). In high-resolution crystal structures of the complete heterotrimeric polymerase, the PA^51-72^-loop can indeed be seen to be located in close proximity of other polymerase domains (13, 14, 16). In the crystal structures of bat influenza A and human influenza B virus polymerases bound to the vRNA promoter, the PA^51-72^-loop is found close to a helical bundle of the C terminus of PB1 (PB1-C) and the N terminus of PB2 (PB2-N) that forms part of the polymerase core (14, 15) (Fig. 1B). However, in the structures of an RNA-free influenza C virus polymerase and an influenza B virus polymerase bound to the 5′ cRNA promoter, the PA^51-72^-loop is found in close proximity to the PB2 NLS (13, 16). Considering the conformational rearrangements that the peripheral polymerase domains can undergo, it is tempting to speculate that the PA^51-72^-loop could play a structural role facilitating or stabilizing certain conformational arrangements.

In this study, we characterize the role of the PA^51-72^-loop in polymerase function using a combination of *in vitro* polymerase activity assays and cell-based vRNP reconstitution assays. We find that the PA^51-72^-loop is not critical for viral transcription but that it plays an essential role during replication of the viral RNA genome. Specifically, we demonstrate that *in vitro* this loop is required for efficient *de novo* replication initiation on both vRNA and cRNA templates and for elongation on a cRNA template.

## RESULTS

### The PA^51-72^-loop is required for polymerase activity.

To address whether the PA^51-72^-loop is required for catalytic functions of the influenza A virus polymerase, a plasmid encoding the PA gene derived from influenza A/WSN/33 (H1N1) virus was generated in which we replaced residues 51 to 72 in the N-terminal endonuclease domain with a GGS-linker (PA^Δ51-72^) (Fig. 1C). Since the PA^51-72^-loop is thought to play a structural role (17), we first determined whether the mutant PA subunit is expressed and whether it can form complete heterotrimeric polymerase complexes. All three polymerase subunits were coexpressed in human HEK-293T cells and purified from cell lysates using a protein A affinity tag on PB2. Silver staining and Western blot analysis confirmed that the truncated PA subunit was expressed and was incorporated into a heterotrimeric polymerase complex together with the PB1 and PB2 subunits (Fig. 1D). A catalytically inactive polymerase with an active-site mutation in the PB1 subunit (PB1a; D445A/D446A) was used as a control (19).

Having shown that the expression and assembly of the truncated polymerase were not affected, we next analyzed the effect of the PA^51-72^-loop deletion on polymerase activity. Viral RNPs were reconstituted by coexpression of the three polymerase subunits, NP, and segment 6 vRNA in human HEK-293T cells, and the accumulation of positive- and negative-sense viral RNA was analyzed by primer extension. The catalytically inactive polymerase (PB1a) was used as a negative control (19). In these minireplicon assays, RNPs containing the PA^Δ51-72^ subunit were fully restricted compared to wild-type RNPs, producing only background levels of mRNA, cRNA, and vRNA (Fig. 1E). Together, these results show that although the PA^51-72^-loop is not required for polymerase heterotrimer assembly, it is essential for polymerase activity.

### The PA^51-72^-loop is dispensable for viral transcription but is essential for RNA replication.

**FIG 2:**
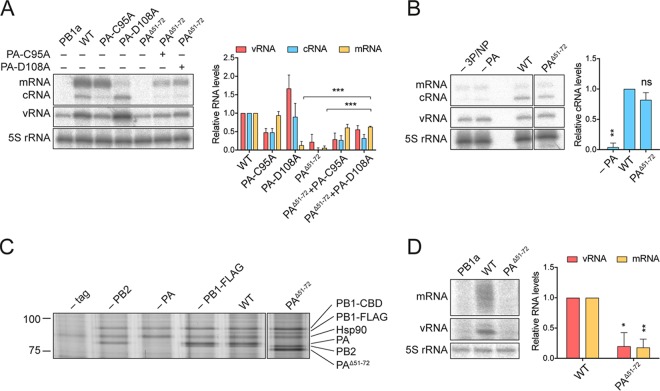
The PA^51-72^-loop is required for replication but not transcription of viral RNPs. (A) HEK-293T cells were cotransfected with plasmids expressing the viral polymerase subunits, NP, and segment 6 vRNA. Where indicated, the polymerase was *trans*-complemented with a replication-deficient (PA-C95A) or a transcription-deficient (PA-D108A) polymerase. The accumulation of viral RNA at 48 h posttransfection was analyzed by primer extension and 6% PAGE. (B) Polymerase subunits (3P) and NP were transiently coexpressed in HEK-293T cells prior to infection with influenza A/WSN/33 (H1N1) virus at an MOI of 5 in the presence of actinomycin D. The accumulation of viral RNA at 6 h postinfection was analyzed by primer extension and 6% PAGE. (C) HEK-293T cells were cotransfected with plasmids expressing PB1-TAP, PB1-FLAG, PB2, and PA as indicated. Heterotrimeric polymerase complexes were purified by IgG Sepharose chromatography. Purified protein complexes were analyzed by SDS-PAGE and silver staining. Molecular weight markers are indicated in kilodaltons. (D) HEK-239T cells were cotransfected with plasmids expressing the viral polymerase and a 47-nucleotide-long RNA-based segment 6 vRNA. The accumulation of viral RNA at 24 h posttransfection was analyzed by primer extension and 12% PAGE. The graphs show the mean signal intensity relative to that of wild-type polymerase from three independent biological replicates (*n* = 3). In panels A and D, mean intensities were corrected for background intensities observed in the negative control (PB1a). Error bars represent standard deviations, and asterisks represent a significant difference in activity of the PA^Δ51-72^ mutant compared to that of wild-type polymerase (two-tailed one sample *t* test), unless otherwise indicated, as follows: *, *P* < 0.05; **, *P* < 0.01; ***, *P* < 0.001; ns, not significant.

To evaluate the effect of the PA^Δ51-72^ mutation on polymerase activity further, the mutant polymerase was *trans*-complemented with either a transcription-deficient (PA-D108A) or a replication-deficient (PA-C95A) polymerase, and the accumulation of viral RNAs in a minigenome assay was analyzed as described above (Fig. 2A). In the absence of *trans*-complementing polymerase, the PA^Δ51-72^ mutant polymerase showed no detectable activity, and when it was *trans*-complemented with the replication-deficient polymerase, no increase in RNA levels was observed compared to levels when only the replication-deficient polymerase was present. In contrast, *trans*-complementation of the PA^Δ51-72^ polymerase with the transcription-deficient polymerase resulted in significant accumulation of viral mRNA, indicating that the PA^Δ51-72^ mutant polymerase is competent in transcription. These results show that the lack of mRNA transcription by the PA^Δ51-72^ polymerase is caused by defective replication and a reduced availability of segment 6 vRNA as a template for viral transcription.

Having shown that the PA^51-72^-loop is dispensable for viral transcription, we next analyzed its role in replication of the viral RNA genome. Viral genome replication is a two-step process, requiring synthesis of an intermediate cRNA template. In a previous study, it has been demonstrated that the intermediary cRNA is detectable in an infection only if viral polymerase is present to stabilize it (19). To test whether the PA^Δ51-72^ polymerase can stabilize nascent cRNA produced during the first step of replication, NP, PB2, and a catalytically inactive (PB1a) polymerase subunit were coexpressed in human HEK-293T cells prior to infection with influenza A/WSN/33 (H1N1) virus in the presence of the cellular transcription inhibitor actinomycin D. Both wild-type and truncated PA^Δ51-72^ polymerases were able to stabilize similar amounts of cRNA, while much lower levels of cRNA were detected in the absence of preexpressed polymerase and NP or if PA was omitted (Fig. 2B). These results show that the PA^51-72^-loop is not needed for the polymerase to bind cRNA.

It has been proposed that polymerase dimerization is required for RNA genome replication (8, 12, 20–22). To address whether the PA^51-72^-loop plays a role in polymerase dimerization, we used a modified version of a previously described assay to probe for polymerase heterotrimer dimerization (21). We coexpressed heterotrimeric polymerase complexes with either a protein A affinity tag or a FLAG tag on PB1 in HEK-293T cells and purified polymerase from cell lysates using the protein A affinity tag. Silver staining analysis revealed that heterotrimers containing PB1-FLAG copurified with heterotrimers containing protein A tag on PB1. Omitting PB2 or PA resulted in no copurification of PB1-FLAG with protein A-tagged PB1, indicating that the full heterotrimer is required for polymerase dimerization. Replacing wild-type PA with PA^Δ51-72^ did not affect the copurification of PB1-FLAG, indicating that the PA^51-72^-loop does not play a role in polymerase heterotrimer dimerization (Fig. 2C).

An interaction between the N terminus of the PA endonuclease and viral NP has been reported (23), and it has been proposed that the PA endonuclease domain is involved in mediating polymerase-NP interactions in vRNPs (15). NP is an important factor in both viral transcription and replication. Template-associated NP represents an elongation factor promoting polymerase processivity by providing structural support for the template RNA during both transcription and replication (24). However, additionally, during replication it is also required for the assembly of the replicative intermediate cRNPs and progeny vRNPs. To address whether the PA^51-72^-loop contributed to polymerase-NP interactions during replication, we took advantage of a previously established NP-independent transcription and replication assay using a truncated 47-nt-long vRNA segment as the template (24). We coexpressed polymerase subunits and a segment 6-based 47-nt-long vRNA in the absence of NP and analyzed the accumulation of viral RNAs. As observed in the minireplicon assays above, polymerase with the truncated PA subunit was unable to produce vRNA and viral mRNA (Fig. 2D). These results show that the PA^51-72^-loop is required for activity in an NP-independent manner, suggesting that it mediates an interaction with a factor other than NP.

Together, these results demonstrate that the PA^51-72^-loop is not involved in viral mRNA synthesis, but it has an essential function in viral replication, which is independent of binding nascent cRNA products, polymerase heterotrimer dimerization, and NP-polymerase interaction.

### The PA^51-72^-loop is required for *de novo* replication initiation.

**FIG 3:**
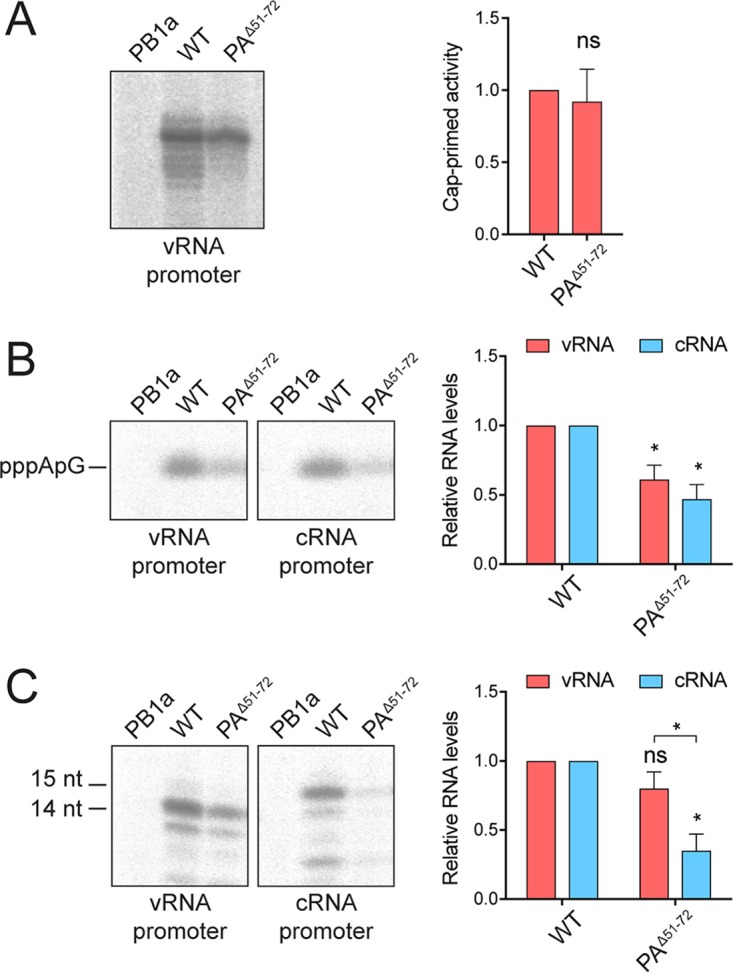
The PA^51-72^-loop is required for *de novo* replication initiation and vRNA synthesis. (A) Purified polymerase was incubated in reaction mixtures containing ATP, UTP, CTP, (α-^32^P]GTP, β-globin mRNA, and vRNA promoter RNA. Reaction products were analyzed by 20% PAGE. [B] Purified polymerase was incubated in reaction mixtures containing ATP, [α-^32^P)GTP, and vRNA or cRNA promoter RNA. Reaction products were treated with alkaline phosphatase before being analyzed by 20% PAGE. (C) Purified polymerase was incubated in reaction mixtures containing ATP, UTP, CTP, [α-^32^P]GTP, ApG primers, and vRNA or cRNA promoter RNA. Reaction products were analyzed by 20% PAGE. The graphs show the mean signal intensity relative to that of wild-type polymerase from three independent biological replicates (*n* = 3) and corrected for background intensities observed in the negative control (PB1a). Error bars represent standard deviations, and asterisks represent a significant difference in activity of the PA^Δ51-72^ mutant compared to that of wild-type polymerase (two-tailed two sample *t* test), unless otherwise indicated, as follows: *, *P* < 0.05; ns, not significant.

The findings above demonstrate that the PA^51-72^-loop is required for viral RNA replication but not cRNA binding. To investigate further at what stage of replication the PA^51-72^-loop is involved, purified recombinant polymerases (Fig. 1D) were used for *in vitro* polymerase activity assays. We first tested the ability of recombinant polymerase to carry out cap-dependent transcription (Fig. 3A). Purified recombinant polymerase was bound to vRNA promoter and incubated in the presence of β-globin mRNA as a cap donor and nucleotides. While the wild-type polymerase was able to cleave the capped β-globin mRNA and use the resulting short capped RNA as a primer for cap-dependent transcription, the PB1a polymerase containing an active-site mutation in the PB1 subunit did not show any detectable activity. However, the PA^Δ51-72^ polymerase was able to carry out cap-dependent transcription without significant loss of activity, in agreement with our results above. To investigate the role of the PA^51-72^-loop in replication, we first asked whether the PA^Δ51-72^ mutant polymerase can carry out primer-independent *de novo* replication initiation. Recombinant polymerase was bound to promoter RNA and incubated in the presence of ATP and GTP (Fig. 3B). The PA^Δ51-72^ polymerase showed significantly decreased activity in synthesizing the initiating pppApG dinucleotide on both vRNA and cRNA templates compared to activity of the wild-type polymerase. No activity was observed with the PB1a active-site mutant. In contrast to results shown in Fig. 1E, where deletion of the PA^51-72^-loop resulted in complete loss of function, *in vitro* polymerase activity was reduced but not abolished. This can be explained by the fact that any defects observed *in vitro* are likely amplified in a cellular context where replication products serve as templates for further rounds of replication. Next, we analyzed the ability of the polymerase to elongate an ApG dinucleotide. To this end, purified polymerase was bound to vRNA or cRNA promoter and incubated in the presence of ApG and nucleotides (Fig. 3C). On the vRNA template, the PA^Δ51-72^ polymerase showed a slightly, but not significantly, reduced ability to elongate ApG, while on the cRNA template, ApG-primed vRNA synthesis by the PA^Δ51-72^ polymerase was significantly impaired compared to the level of the wild-type polymerase. Together, these data confirm that the PA^51-72^-loop is not involved in cap snatching and the initiation of viral transcription but that it is important for several processes during viral replication. Specifically, our data show that the PA^51-72^-loop is required for *de novo* replication initiation on both vRNA and cRNA templates. Interestingly, it is also required for elongation on the cRNA template but appears less important for elongation on a vRNA template.

## DISCUSSION

In this study, we aimed to characterize the function of a surface-exposed flexible loop in the endonuclease domain of the PA polymerase subunit of the influenza A virus heterotrimeric polymerase complex. We found that this flexible PA^51-72^-loop is not required for heterotrimeric polymerase assembly and that in a cellular context a polymerase lacking the PA^51-72^-loop is able to transcribe but unable to replicate viral RNA. Furthermore, the PA^Δ51-72^ polymerase was able to stabilize nascent cRNA produced by replicating vRNPs when provided *in trans*. Accordingly, recombinant polymerase lacking the PA^51-72^-loop was able to carry out cap snatching and capped RNA primer-dependent transcription but was restricted in its ability to initiate replication *de novo*. Elongation of the initiating dinucleotide was significantly more restricted on a cRNA template than on a vRNA template.

Our finding that the PA^51-72^-loop is not required for viral transcription is consistent with a previous study reporting that an isolated PA endonuclease domain lacking the PA^51-72^-loop was active in endonucleolytic cleavage of RNA *in vitro* (17). Here, we not only confirm these previous results but also provide evidence for cap-dependent transcription *in vitro* as well as in a cellular context using complete heterotrimeric polymerase complexes. These results are fully consistent with the structural analysis of the PA endonuclease domain showing that residues 51 to 72 form a surface-exposed loop which is distinctly separate from the endonuclease active site and therefore not expected to be involved in endonuclease activity during cap snatching (5, 7).

We also show that the polymerase lacking the PA^51-72^-loop is restricted in primer-independent initiation *in vitro* on both vRNA and cRNA templates, indicating a role of the PA^51-72^-loop in replication initiation. This is surprising since residues 51 to 72 of the PA subunit are surface exposed and located far away from the polymerase active site and the template entry channel (13–16). It is therefore difficult to envisage a direct active role of the PA^51-72^-loop in RNA synthesis. It is more likely that the PA^51-72^-loop is involved in stabilizing a replication-competent conformation of the polymerase by associating with other parts of the polymerase. The PA^51-72^-loop makes different interactions with the rest of the polymerase, depending on the conformational arrangement of its flexible domains (Fig. 1B). In the transcription-ready conformation it interacts with a helical bundle of the C terminus of PB1 and the N terminus of PB2, while in the cRNA-bound and apo conformations it is close to the PB2 627/NLS domains. Currently, the conformation of the influenza virus replicase remains obscure, and therefore it is difficult to assess how important these interactions might be for replication. Alternatively, the PA^51-72^-loop could be mediating an interaction with another viral or cellular factor required for RNA genome replication. Indeed, the PA endonuclease domain has previously been implicated in an interaction with NP (23), and fitting of the polymerase structure into the cryo-EM structure of the vRNP suggests that the PA endonuclease makes contacts with NP in vRNPs (15). However, our finding that vRNPs are able to carry out transcription, which presumably also requires fully assembled vRNPs, argues against a critical role of the PA^51-72^-loop in mediating an interaction with NP in the vRNP. Furthermore, we found that the PA^Δ51-72^ mutant is unable to replicate a 47-nt-long vRNA template that does not require NP for replication, suggesting an NP-independent role for the PA^51-72^-loop in RNA genome replication.

It has been proposed that viral genome replication requires multiple viral polymerases (8, 12). Specifically, it has been shown that vRNPs and cRNPs are *trans*-activated by RNA-free polymerase *in vitro* (8). It is likely that *trans*-activation occurs through a direct interaction between the RNP-resident polymerase and an RNA-free *trans*-activating polymerase. A direct interaction between viral polymerases could be an efficient mechanism to facilitate transfer of the nascent viral RNA replication product from the replicating RNP to an RNA-free polymerase to initiate the coreplicative assembly of the replication product into a new RNP, as well as to facilitate any potential *trans*-acting or *trans*-activating functions of the polymerase (8, 12). Indeed, oligomerization of the influenza A virus polymerase has been reported (20, 21) with the PA C-terminal region contributing to the dimerization interface (20). The PA^51-72^-loop falls outside this previously identified region, and, in agreement, deletion of the PA^51-72^-loop did not affect polymerase dimerization in mammalian cells. However, it should be noted that several dimerization interfaces might exist, corresponding to different functional states of a replicating polymerase, influenced by the nature of RNA bound to the polymerase (13, 16, 20). Therefore, we cannot exclude the possibility that the PA^51-72^-loop is involved in mediating polymerase oligomerization through an alternative oligomerization interface.

Numerous cellular factors have been identified interacting with the viral polymerase (4, 25, 26), and the PA^51-72^-loop might also play a role in recruiting a cellular factor to the polymerase. Recently, ANP32A has been identified as a cellular factor underlying the PB2 amino acid residue 627-mediated host restriction of the influenza virus polymerase (27). ANP32A has been shown to be specifically required for the cRNA-to-vRNA step of replication (28). Interestingly, our results show that, in addition to replication initiation on both vRNA and cRNA templates, elongation on the cRNA template is specifically affected by the deletion of the PA^51-72^-loop. Further studies will be needed to address whether the PA^51-72^-loop could be involved in promoting replication in an ANP32A-dependent manner.

The influenza B virus polymerase contains a PA-loop similar in length and sequence to that present in the influenza A virus polymerase (Fig. 1A), suggesting that the PA-loop performs the same function in both types of polymerases. Intriguingly, in the influenza C virus polymerase, a truncated form of the PA-loop is present. As our data on the influenza A virus polymerase show that the PA-loop is essential for viral RNA genome replication, we can only speculate that in the influenza C virus polymerase this shorter loop is able to carry out this function in replication, possibly because of compensatory changes elsewhere in the polymerase or in another viral factor.

In summary, we demonstrate here that the PA^51-72^-loop of the influenza virus RNA polymerase is needed for the replication of the viral RNA genome, in particular for *de novo* replication initiation on both vRNA and cRNA templates, and vRNA synthesis. However, it is not essential for the capped RNA primer-dependent transcriptional activity of the polymerase. We propose that the PA^51-72^-loop may participate in the stabilization of the replicase conformation of the polymerase. These findings further our understanding of the PA endonuclease domain as well as of the intricate mechanisms of influenza A virus RNA genome replication.

## MATERIALS AND METHODS

### Cells, viruses and plasmids.

Human embryonic kidney 293T (HEK-293T) cells were cultured in Dulbecco's modified Eagle medium (DMEM) supplemented with 10% fetal calf serum (FCS). Madin-Darby bovine kidney (MDBK) epithelial cells were cultured in minimal essential medium (MEM) supplemented with 10% FCS and 2 mM l-glutamine. Cells were maintained at 37°C and 5% CO_2_. Recombinant influenza A/WSN/33 (H1N1) virus was generated using the pHW2000 eight-plasmid system (29). Plasmids pcDNA-NP, pcDNA-PA, pcDNA-PB1, pcDNA-PB2, pcDNA-3a (30), pcDNA-PB1-FLAG, pcDNA-PB1-TAP (PB1 fused to a C-terminal tandem affinity purification [TAP] tag that consists of a calmodulin binding domain [CBD], a tobacco etch virus [TEV] protease cleavage site, and two copies of protein A) (31), pcDNA-PB2-TAP (32), pcDNA-PA-C95A, pcDNA-PA-D108A (33), pcDNA-PB1a (19), pPOLI-NA (34), and pPOLI-NA47 (35) have been described previously. The plasmid pcDNA-PA^Δ51-72^ was generated from pcDNA-PA using site-directed PCR mutagenesis.

### Purification of recombinant influenza virus polymerase.

For purifying polymerase for assessing polymerase heterotrimer assembly and *in vitro* activity assays, approximately 5.5 × 10^6^ HEK-293T cells were transiently transfected in 10-cm dishes with 5 μg each of pcDNA-PB2-TAP, pcDNA-PB1/pcDNA-PB1a, and pcDNA-PA/pcDNA-PA^Δ51-72^ using Lipofectamine 2000 reagent (Invitrogen) and Opti-MEM (Invitrogen) according to the manufacturer's instructions. For polymerase heterotrimer dimerization assays, HEK-293T cells were transfected with 3 μg each of pcDNA-PB1-TAP, pcDNA-PB1-FLAG, pcDNA-PB2, and pcDNA-PA/pcDNA-PA^Δ51-72^, as above. Cells were harvested at 48 h posttransfection, lysed in 500 μl of Tris lysis buffer (50 mM Tris-HCl [pH 8.0], 200 mM NaCl, 25% glycerol, 0.5% Igepal CA-630, 1 mM dithiothreitol [DTT], 1 mM phenylmethylsulfonyl fluoride [PMSF], 1× complete EDTA-free protease inhibitor cocktail tablet [Roche]) at 4°C for 1 h, and centrifuged at 17,000 × *g* for 5 min. The cleared cell lysate was diluted 1:5 in binding buffer (20 mM Tris-HCl [pH 8.0], 150 mM NaCl) and incubated with 50 μl of washed IgG Sepharose (GE Healthcare) at 4°C for 3 h. After binding, the IgG Sepharose beads were washed three times in wash buffer (10 mM Tris-HCl [pH 8.0], 150 mM NaCl, 10% glycerol, 0.1% Igepal CA-630, 1 mM PMSF). Recombinant polymerase was released using tobacco etch virus (AcTEV) protease in elution buffer (10 mM Tris-HCl [pH 8.0], 150 mM NaCl, 10% glycerol, 0.1% Igepal CA-630, 1 mM DTT, 1 mM PMSF, 1× complete EDTA-free protease inhibitor cocktail tablet) at 4°C overnight and cleared from IgG Sepharose by centrifugation at 17,000 × *g* for 5 min 4°C. Purified polymerase complexes were analyzed by SDS-PAGE, followed by silver staining (SilverXpress; Invitrogen) or Western blotting using polyclonal rabbit antibodies raised against PA (32), PB1 (GTX125923), and PB2 (36) and a horseradish peroxidase (HRP)-conjugated goat anti-rabbit IgG secondary antibody (Sigma-Aldrich) and an Immobilon Western chemiluminescence HRP substrate kit (Millipore) for detection.

### RNP reconstitution and primer extension analysis.

Viral RNPs were reconstituted by transiently transfecting approximately 1 × 10^6^ HEK-293T cells in 35-mm dishes with 1 μg each of pcDNA-PA/pcDNA-PA^Δ51-72^, pcDNA-PB1/pcDNA-PB1a, pcDNA-PB2, pcDNA-NP, and pPOLI-NA using Lipofectamine 2000 and Opti-MEM according to the manufacturer's instructions. Cells were harvested 24 h posttransfection. For *trans*-complementing RNP reconstitutions, approximately 1 × 10^6^ HEK-293T cells in 35-mm dishes were transfected as before with 1 μg each of pcDNA-PA^Δ51-72^, pcDNA-PB1/pcDNA-PB1a, pcDNA-PB2, pcDNA-NP, and pPOLI-NA and, as indicated in Fig. 2A, with 1 μg of *trans*-complementing pcDNA-PA-C95A or pcDNA-D108A or empty vector pcDNA-3a control. Cells were harvested 48 h posttransfection. Total RNA was extracted using Tri reagent (Sigma-Aldrich) and dissolved in 20 μl of double-distilled water. The accumulation of viral mRNA, cRNA, and vRNA was analyzed by primer extension using ^32^P-labeled primers specific for negative- or positive-sense segment 6 RNA as well as 5S rRNA (35, 37). 5S rRNA was used as an internal loading control. Primer extension products were analyzed by 6 to 12% denaturing PAGE with 7 M urea in Tris-borate-EDTA (TBE) buffer and detected by autoradiography. ImageJ was used to analyze the ^32^P-derived signal (38).

### cRNA stabilization assay.

The ability of polymerase to stabilize nascent cRNA products was analyzed as previously described (39). Approximately 1 × 10^6^ HEK-293T cells were transiently transfected in 35-mm dishes with 1 μg each of pcDNA-PA/pcDNA-PA^Δ51-72^, pcDNA-PB1a, pcDNA-PB2, and pcDNA-NP using Lipofectamine 2000 and Opti-MEM according to the manufacturer's instructions. At 48 h posttransfection, cells were infected with influenza A/WSN/33 (H1N1) virus at a multiplicity of infection (MOI) of 5 in DMEM containing 0.5% FCS in the presence of 5 μg/ml of actinomycin D for 1 h at room temperature. Cells were incubated at 37°C for 6 h before cells were harvested, and total RNA was extracted. The accumulation of viral RNA was analyzed by primer extension as described above.

### *In vitro* transcription assay.

The ability of purified recombinant polymerase to use β-globin mRNA as a source of capped RNA primer for cap-dependent transcription was assessed as described previously (40). Reaction mixtures containing 0.05 μM [α-^32^P]GTP (3,000 Ci/mmol; Perkin-Elmer), 1 mM ATP, 0.5 mM CTP, 0.5 mM UTP, 50 ng of rabbit β-globin mRNA (Sigma-Aldrich), 5 mM MgCl_2_, 1 mM DTT, 2 U/μl RNasin, 0.5 μM 5′ vRNA promoter, 0.5 μM 3′ vRNA promoter, and 10 ng of recombinant polymerase were incubated at 30°C for 12 h. Reactions were terminated by incubation at 95°C for 3 min, addition of an equal volume of 80% formamide, 1 mM EDTA bromophenol blue, and xylene cyan, and further incubation at 95°C for 3 min. Reaction products were resolved by 20% denaturing PAGE containing 7 M urea in TBE buffer and visualized by autoradiography. ImageJ was used to analyze the ^32^P-derived signal (38).

### *In vitro* dinucleotide replication initiation assay.

The ability of purified recombinant polymerase to synthesize a pppApG dinucleotide *de novo* was assessed as described before (11). Reaction mixtures containing 0.05 μM [α-^32^P]GTP (3,000 Ci/mmol; Perkin-Elmer), 1 mM ATP, 5 mM MgCl_2_, 1 mM DTT, 2 U/μl RNasin, 0.5 μM 5′ vRNA or cRNA promoter, 0.5 μM 3′ vRNA or cRNA promoter, and 10 ng of recombinant polymerase were incubated at 30°C for 12 h, followed by heating to 95°C for 3 min. Reaction mixtures were treated with 1 U of FastAP thermosensitive alkaline phosphatase (Thermo Scientific) for 1 h at 37°C. Reactions were terminated by addition of an equal volume of 80% formamide, 1 mM EDTA, bromophenol blue, and xylene cyan and incubation at 95°C for 3 min. Reaction products were resolved by 20% denaturing PAGE containing 7 M urea in TBE buffer and visualized by autoradiography. ImageJ was used to analyze the ^32^P-derived signal (38).

### *In vitro* ApG extension assay.

The ability of purified recombinant polymerase to extend an ApG dinucleotide was assessed as described previously (9). Reaction mixtures containing 0.05 μM [α-^32^P]GTP (3,000 Ci/mmol; Perkin-Elmer), 1 mM ATP, 0.5 mM CTP, 0.5 mM UTP, 0.25 mM ApG (IBA Lifesciences), 5 mM MgCl_2_, 1 mM DTT, 2 U/μl RNasin, 0.5 μM 5′ vRNA or cRNA promoter, 0.5 μM 3′ vRNA or cRNA promoter, and 10 ng of recombinant polymerase were incubated at 30°C for 12 h. Reactions were terminated by incubation at 95°C for 3 min, addition of an equal volume of 80% formamide, 1 mM EDTA bromophenol blue, and xylene cyan, and further incubation at 95°C for 3 min. Reaction products were resolved by 20% denaturing PAGE containing 7 M urea in TBE buffer and visualized by autoradiography. ImageJ was used to analyze the ^32^P-derived signal (38).
